# Comprehensive analysis of ceRNA network related to lincRNA in glioblastoma and prediction of clinical prognosis

**DOI:** 10.1186/s12885-021-07817-5

**Published:** 2021-01-26

**Authors:** Guangdong Liu, Danian Liu, Jingjing Huang, Jianxin Li, Chuang Wang, Guangyao Liu, Shiqiang Ge, Haidong Gong

**Affiliations:** 1 Department of Neurosurgery, Hongqi Hospital Affiliated to Mudanjiang Medical University, No. 5, Tongxiang Road, Aimin, MuDanJiang, HeiLongJiang China; 2 Department of Neurology, Hongqi Hospital Affiliated to Mudanjiang Medical University, MuDanJiang, China; 3 Department of Infectious Diseases, Hongqi Hospital Affiliated to Mudanjiang Medical University, MuDanJiang, China; 4 Department of Neurosurgery, Jiaozuo People’s Hospital, JiaoZuo, China

**Keywords:** Glioblastoma, ceRNA, lincRNA, Weighted gene co-expression network analysis, Prognostic model

## Abstract

**Background:**

Long intergenic non-coding RNAs (lincRNAs) are capable of regulating several tumours, while competitive endogenous RNA (ceRNA) networks are of great significance in revealing the biological mechanism of tumours. Here, we aimed to study the ceRNA network of lincRNA in glioblastoma (GBM).

**Methods:**

We obtained GBM and normal brain tissue samples from TCGA, GTEx, and GEO databases, and performed weighted gene co-expression network analysis and differential expression analysis on all lincRNA and mRNA data. Subsequently, we predicted the interaction between lincRNAs, miRNAs, and target mRNAs. Univariate and multivariate Cox regression analyses were performed on the mRNAs using CGGA data, and a Cox proportional hazards regression model was constructed. The ceRNA network was further screened by the DEmiRNA and mRNA of Cox model.

**Results:**

A prognostic prediction model was constructed for patients with GBM. We assembled a ceRNA network consisting of 18 lincRNAs, 6 miRNAs, and 8 mRNAs. Gene Set Enrichment Analysis was carried out on four lincRNAs with obvious differential expressions and relatively few studies in GBM.

**Conclusion:**

We identified four lincRNAs that have research value for GBM and obtained the ceRNA network. Our research is expected to facilitate in-depth understanding and study of the molecular mechanism of GBM, and provide new insights into targeted therapy and prognosis of the tumour.

## Background

Glioblastoma (GBM) is the most aggressive and destructive primary malignant central nervous system tumour [1]. At present, the most established treatment protocol for this tumour involves curative surgery and adjuvant radiotherapy combined with temozolomide; however, its prognosis is still very poor, and the average survival time of patients is approximately 2 years [2]. Moreover, various treatment methods affect normal brain tissue, and the quality of life of patients does not significantly improve afterwards. Therefore, an in-depth study of the molecular mechanism of GBM occurrence and development, while exploring possible therapeutic targets, may prove beneficial for the diagnosis and treatment of the tumour.

Although approximately 90% of genes in the human genome can be transcribed, only approximately 2% of the genes are protein coding, and non-coding RNAs account for most of the remainder [3]. Long non-coding RNAs (lncRNAs) contain > 200 nucleotides and have no protein coding function. They are mainly transcribed from different regions of the genome by RNA polymerase II, and have been shown to be closely related to cancer [4, 5]. The tissue specificity of lncRNA expression has been reported to be higher than that of mRNA expression, which applies even in pathological conditions such as cancer [6]. Long intergenic non-coding RNA (lincRNA) is a type of lncRNA that does not overlap with exons of protein-coding genes and other non-lincRNA genes, and participates in many important biological processes [7, 8]. Cancer-related lincRNAs may be targeted to provide new ways for cancer diagnosis and treatment [9]. Linc-ROR is a tumour promoter, which mainly participates in tumour cell proliferation, apoptosis, invasion, angiogenesis, and cancer stem cell generation by regulating target genes [10]. *NEAT1* is a p53-regulated lincRNA, which plays a key role in cancer occurrence [11]. LincRNA-p21 plays an important role in regulating TAM function in tumour microenvironments, and can be used as a new cancer treatment target for macrophage infiltration [12]. In general, molecular mechanisms related to lincRNA in tumours have important research value.

The COX proportional hazard regression model can analyze the impact of several factors on survival simultaneously, and is the most commonly used multi-factor analysis method for survival analysis. Long et al. found that a prognostic model composed of 4 mRNAs is a reliable tool for predicting the survival of liver cancer patients [13]. A new type of COX model containing 6 genes can improve the prognosis prediction of patients with uterine sarcoma [14]. In summary, the COX model can help clinicians select personalized treatment plans for patients and predict prognosis.

A competitive endogenous RNA (ceRNA) network is the interaction between lncRNAs, miRNAs, and mRNAs that constitutes a complex regulatory network system, which has extensive functions in the human genome and plays a significant role in cancer [15]. The ceRNA network includes mRNAs, miRNAs, lncRNAs, and circRNAs, which play a key role in the occurrence and development of gastric cancer and colorectal cancer, and can be used as biomarkers for tumour treatment [16, 17]. It has been reported that linc-ROR can be used as the ceRNA of miR-145 to regulate the proliferation, invasion, and tumourigenicity of pancreatic cancer cells [18]. Further, a study has demonstrated that lincRNA-HOTAIR is highly expressed in a variety of tumour tissues and cells, and is associated with tumour metastasis and poor prognosis. Additionally, its related ceRNA network has been shown to play a role in the progression of kidney cancer [19]. At present, there are few studies on the ceRNA network in glioblastoma, conducted mainly for a single database and with a small sample, and there are fewer prognostic prediction analyses [20, 21]. In their study, Liu et al. used two databases to construct a ceRNA network for GBM, but did not incorporate WGCNA analysis, nor did they systematically construct a prognostic prediction model [22]. Therefore, in this study, we comprehensively analysed the GBM data retrieved from The Cancer Genome Atlas (TCGA), National Center for Biotechnology Information (NCBI), Gene Expression Omnibus (GEO), Chinese Glioma Genome Atlas (CGGA), and Genotype Tissue Expression (GTEx) databases. We used weighted gene co-expression network analysis (WGCNA) and differential analysis to screen key lincRNAs, construct lincRNA-related ceRNA networks in GBM, and obtain a prognostic model. We believe this study provides a basis for further studies on the molecular mechanism of GBM and exploration of new therapeutic targets.

## Methods

### Data acquisition and pre-processing

**Fig. 1:**
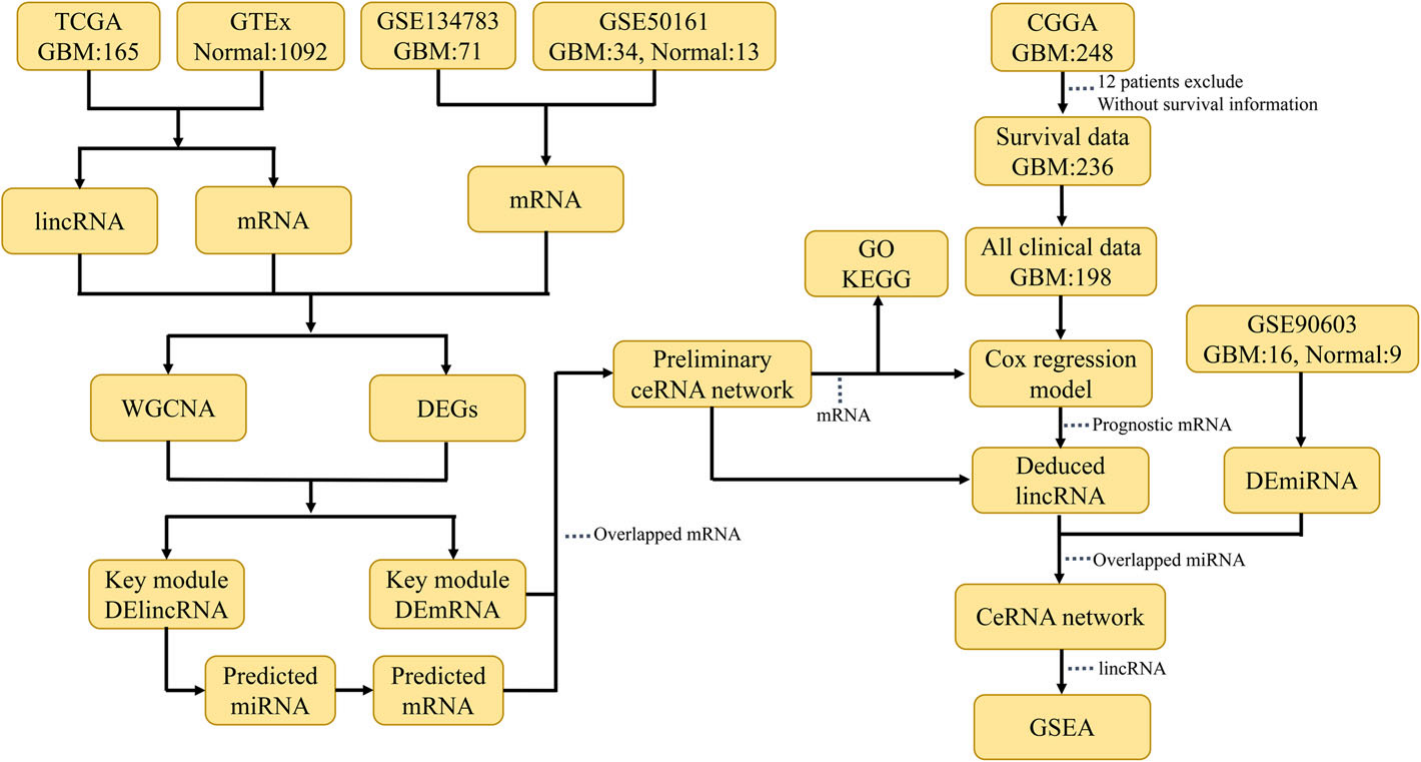
Study workflow. TCGA, The Cancer Genome Atlas; GTEx, Genotype Tissue Expression; GEO, Gene Expression Omnibus; CGGA, Chinese Glioma Genome Atlas; GBM, glioblastoma; WGCNA, Weighted Gene Co-expression Network Analysis; DEG, differentially expressed genes; GO, Gene Ontology; KEGG, Kyoto Encyclopedia of Genes and Genomes; GSEA, Gene Set Enrichment Analysis

Figure 1 shows the flow diagram of our study. We obtained RNA-Seq data of 165 GBM samples and 1029 normal brain tissue samples from the TCGA and GTEx databases, respectively, using the UCSC Xena Browser (https://xena.ucsc.edu/). Further, the mRNA-seq and clinical data of 693 glioma samples were retrieved from CGGA (http://cgga.org.cn/), from which 248 GBM samples were selected for this study. Among the selected samples, 236 patients had survival information, while 198 had all clinical information. Additionally, we retrieved GSE50161 (comprising 34 GBM and 13 normal samples; platform: Affymetrix-GPL570), GSE134783 (comprising 71 GBM samples; platform: Affymetrix-GPL570), and GSE90603 (comprising 16 GBM and 9 normal samples; platform: Affymetrix-GPL21572) from the GEO database (https://www.ncbi.nlm.nih.gov/geo/).

The IDs of all samples were transformed into gene symbols based on GENCODE (https://www.gencodegenes.org/human/). The RNA-seq data fetched from TCGA and GTEx were collated and merged, and the expression matrices of mRNA and lincRNA were obtained. We also combined the GEO data and obtained the mRNA and miRNA expression matrix. The unit of RNA-seq data is FPKM. The R package oligo [23] was utilized for format conversion, missing data filling, background correction, and data normalisation. All data were downloaded from public databases, and the data were approved by medical ethics when they were first published. Therefore, this study did not require medical ethics documents.

### Construction of weighted gene co-expression network

Using the combined data of TCGA and GTEx, a weighted gene co-expression network was constructed for lincRNA and mRNA. Based on the mRNA of the GEO combined data, a weighted gene co-expression network was constructed. WGCNA R software package [24] was used to build a co-expression network to mine key modules related to GBM. First, a hierarchical cluster analysis was performed to check the heterogeneity of the samples. Subsequently, according to the scale-free topological standard, we chose the appropriate soft threshold power (β) to construct a weighted adjacency matrix and convert the adjacency relationship into a topological overlap matrix. Finally, we obtained the gene modules, and determined the modules related to the pathogenicity of GBM through the relationship between the modules and traits, and used a scatter plot to show the correlation between gene significance and module membership.

### Screening of differentially expressed genes

We used the Limma R package [25] to screen the differentially expressed lincRNA (DElincRNA) between the GBM patients and control group from the combined data of TCGA and GTEx.

Differential expression analysis was performed on the mRNA data in the TCGA and GTEx combined data and GEO combined data, and the two selected differential genes were combined to obtain differentially expressed mRNA (DEmRNA). Additionally, differential analysis was performed on GSE90603 to obtain DEmiRNA. (*P* < 0.05, and |log2 FC| ≥ 1).

### Preliminary construction of ceRNA network

The most relevant modules to GBM were selected and analysed with DElincRNA to screen for overlapping lincRNA. Further, miRcode (http://www.mircode.org/) was used to predict miRNA, and the miRNA target mRNA was predicted using miRDB (http://www.mirdb.org/), miRTarBase (http://mirtarbase.mbc.nctu.edu.tw/), and TargetScan (http://www.targetscan.org/). The predicted mRNA, WGCNA key module mRNA (TCGA and GTEx, GEO), and DEmRNA were analysed to obtain the overlapping mRNA. Additionally, Cytoscape 3.7.2 software was used to visualise the ceRNA network.

### Gene function and pathway enrichment analysis

We used the clusterProfiler R package [26] for Gene Ontology (GO) analysis and Kyoto Encyclopedia of Genes and Genomes (KEGG) enrichment analysis. GO is used to describe gene function, and KEGG is used to obtain possible pathways. Further, the GOplot R package [27] was used to visualise the GO term or KEGG approach (adjusted *p* < 0.05).

### Construction of cox regression model

Univariate COX regression analysis was performed on the CGGA data, using the Survival R software package, to evaluate the effect of mRNA expression on the survival time of GBM patients. Additionally, we performed multivariate COX regression analysis, constructed a multivariate Cox regression model, and identified the corresponding coefficients of GBM prognostic features. Further, we calculated the risk score to predict survival time, dividing the samples into high-risk and low-risk groups, with the median as the critical value. The “predict ()” function was used to calculate the risk score: risk score = h_0_(t)*exp.(β_1_x_1_ + β_2_x_2_+ … + β_n_x_n_). The correlation between the prognostic characteristics of patients and overall survival rate was then calculated through univariate and multivariate Cox regression analyses of clinical factors related to overall survival. Finally, the R package survivalROC was used to draw the receiver operating characteristic curve and calculate the area under the curve (AUC).

### CeRNA network in GBM

Based on the mRNA obtained through multivariate Cox regression analysis and DEmiRNA, we screened and reconstructed the ceRNA network.

### Gene set enrichment analysis (GSEA)

GSEA software was used to analyse the RNA-seq data of lincRNA retrieved from the CGGA database. According to the median of lincRNA expression, the samples were divided into high and low expression groups. Statistical significance was set at *p* < 0.05. A set of genes “c2. cp. kegg. v7.2. symbols. gmt” was retrieved from the Molecular Signature Database (MSigDB, http://software.broadinstitute.org/gsea/msigdb/index.jsp) and selected as the reference gene set.

## Results

### WGCNA identifies key modules

**Fig. 2:**
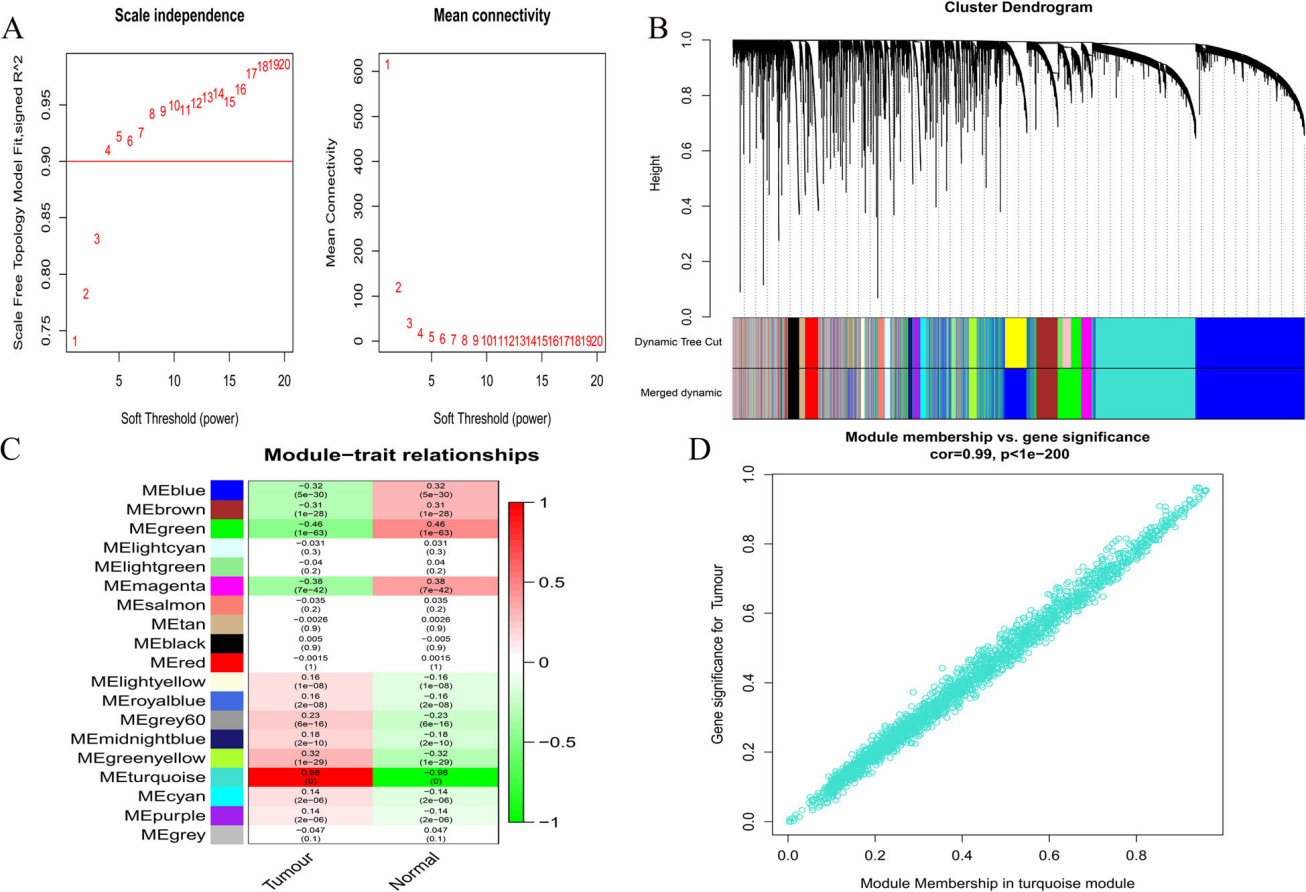
Identification of lincRNAs modules highly related to traits. **a**, Analysis of network topology for various soft-thresholding powers. Left: Analysis of the scale-free fit index for various soft-thresholding powers (β). Right: Analysis of the mean connectivity for various soft-thresholding powers. **b**, Cluster dendrogram of all lincRNA in the co-expression network. As a result, 19 co-expression modules were constructed and was shown in different color. **c**, Module-trait associations of lincRNAs were evaluated by correlations between MEs and GBM pathogenicity. Each row corresponds to a module eigengene, column to a trait. Each cell contains the corresponding correlation and *p*-value. **d**, The correlation between Gene Significance (GS) and Module Membership (MM) in the turquoise module. There is a highly significant correlation between GS and MM

We combined GTEx and TCGA data to construct a co-expression network for all lincRNAs, using the R package ‘WGCNA’, and confirmed that the β value in the network was 4 (Fig. 2a). Further, the dynamic tree cutting method was used to generate co-expression modules, and the closely related modules were merged into larger modules. Finally, 19 modules were generated in the lincRNA co-expression network (Fig. 2b). The module eigengenes (MEs) of the turquoise module had the strongest correlation with GBM traits (Fig. 2c). Figure 2d shows the correlation between gene significance and module membership in the turquoise module, which was considered a key module containing 2206 lincRNAs.

**Fig. 3:**
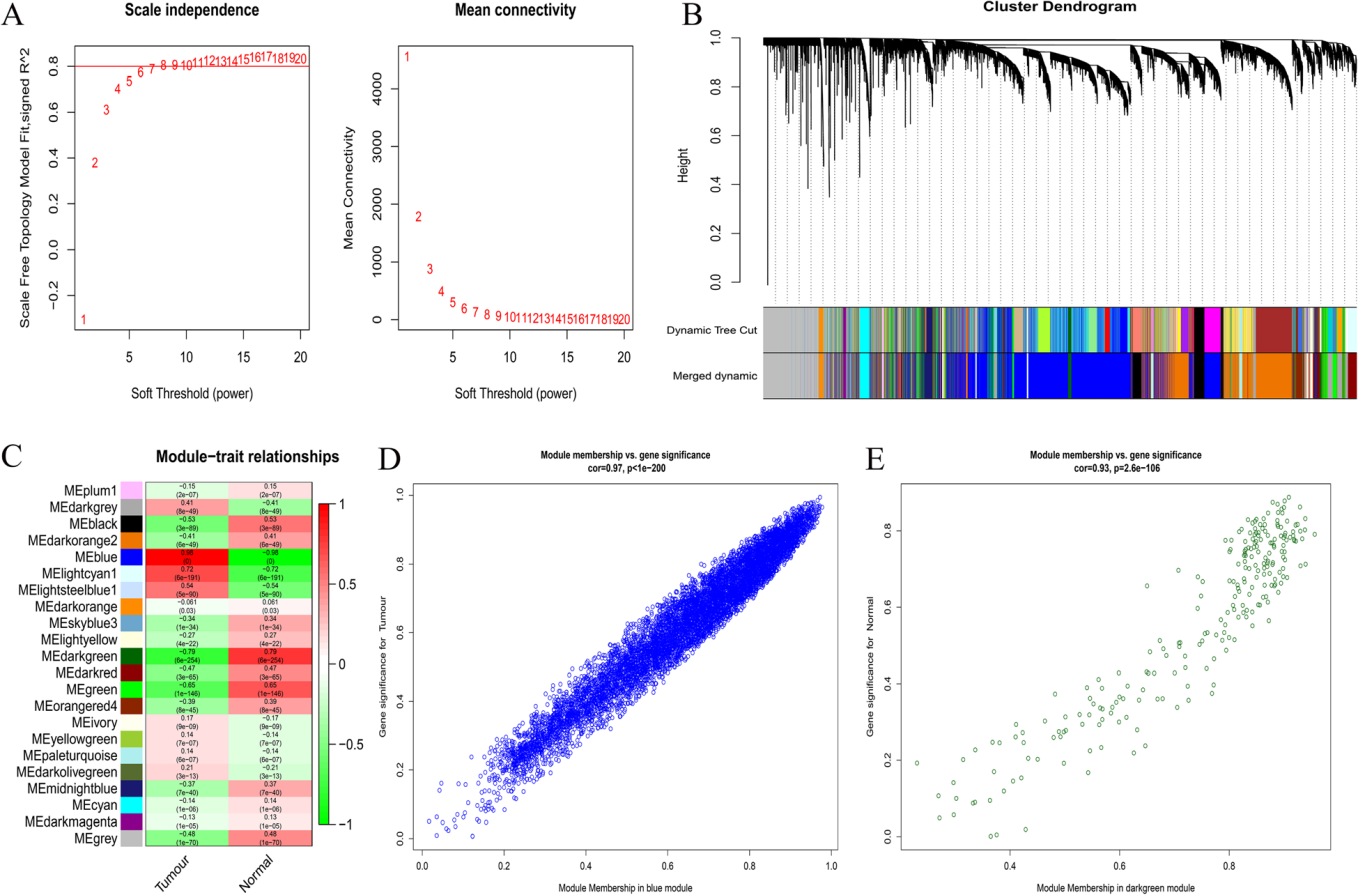
Identification of mRNAs modules highly related to traits in the combined data of TCGA and GTEx. **a**, Determination of soft-thresholding power. **b**, Cluster dendrogram of all mRNA in the co-expression network. **c**, The correlation between modules and GBM pathogenicity were displayed. **d**, The correlation between GS and MM in the blue and darkgreen modules

**Fig. 4:**
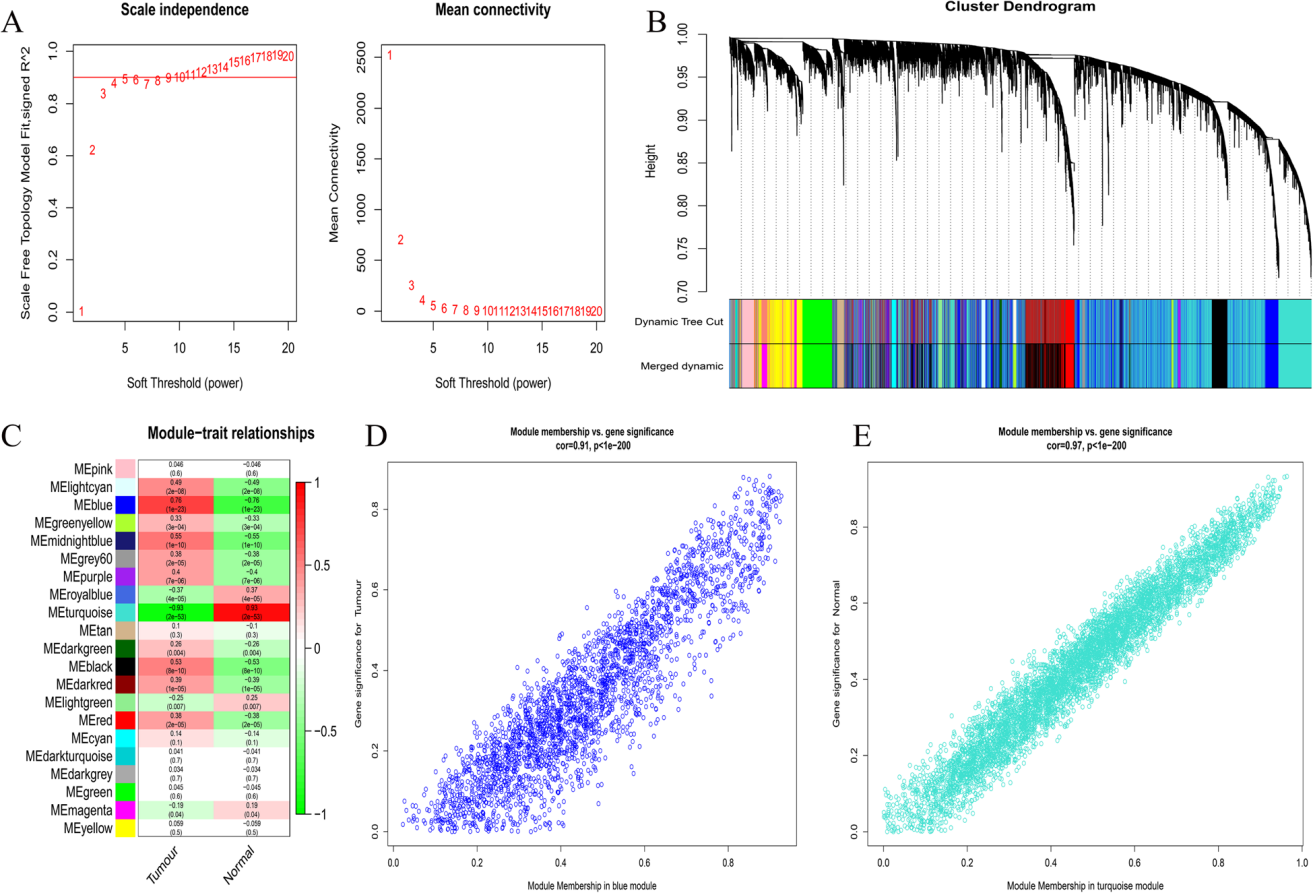
Identification of mRNAs modules highly related to traits in the combined data of GEO. **a**, Determination of soft-thresholding power. **b**, Cluster dendrogram of all mRNA in the co-expression network. **c**, The correlation between modules and GBM pathogenicity were displayed. **d**, The correlation between GS and MM in the blue and turquoise modules

We also constructed the co-expression networks of all mRNAs for GTEx and TCGA combined data and GEO combined data, comprising 20,270 and 14,807 mRNAs, respectively. In the mRNA co-expression networks of the two sets of data, β values were 8 and 4 (Fig. 3a and Fig. 4a), and 22 and 21 modules were generated (Fig. 3b and Fig. 4b), respectively. In the combined GTEx and TCGA data, the MEs of the blue and dark green modules have the strongest correlations with the tumour and normal traits, respectively (Fig. 3c). The blue and dark green modules were considered key modules, comprising 7736 mRNAs. In the GEO data, Fig. 4c shows that the MEs of the blue and turquoise modules (comprising 8022 mRNAs) have the strongest correlations with the tumour and normal traits, respectively. Figures 3d-e and Fig. 4d-e show the correlation between gene significance and module membership.

### Identification of differentially expressed lincRNAs (DElincRNAs), miRNAs (DEmiRNAs) and mRNAs (DEmRNAs)

We identified DElincRNAs and DEmRNAs from the GTEx and TCGA combined data, including 163 up-regulated lincRNAs, 176 down-regulated lincRNAs, 2953 up-regulated mRNAs, and 2932 down-regulated mRNAs. From the GEO data, 2434 up-regulated and 1995 down-regulated mRNAs were identified. The two groups of mRNAs were then comprehensively analysed to obtain overlapping 1040 down-regulated mRNAs and 1358 up-regulated mRNAs. Finally, 339 DElincRNAs and 2398 DEmRNAs were identified. Through the GSE90603 dataset, 155 down-regulated and 134 up-regulated miRNAs were identified.

### Preliminary construction of ceRNA network

**Fig. 5:**
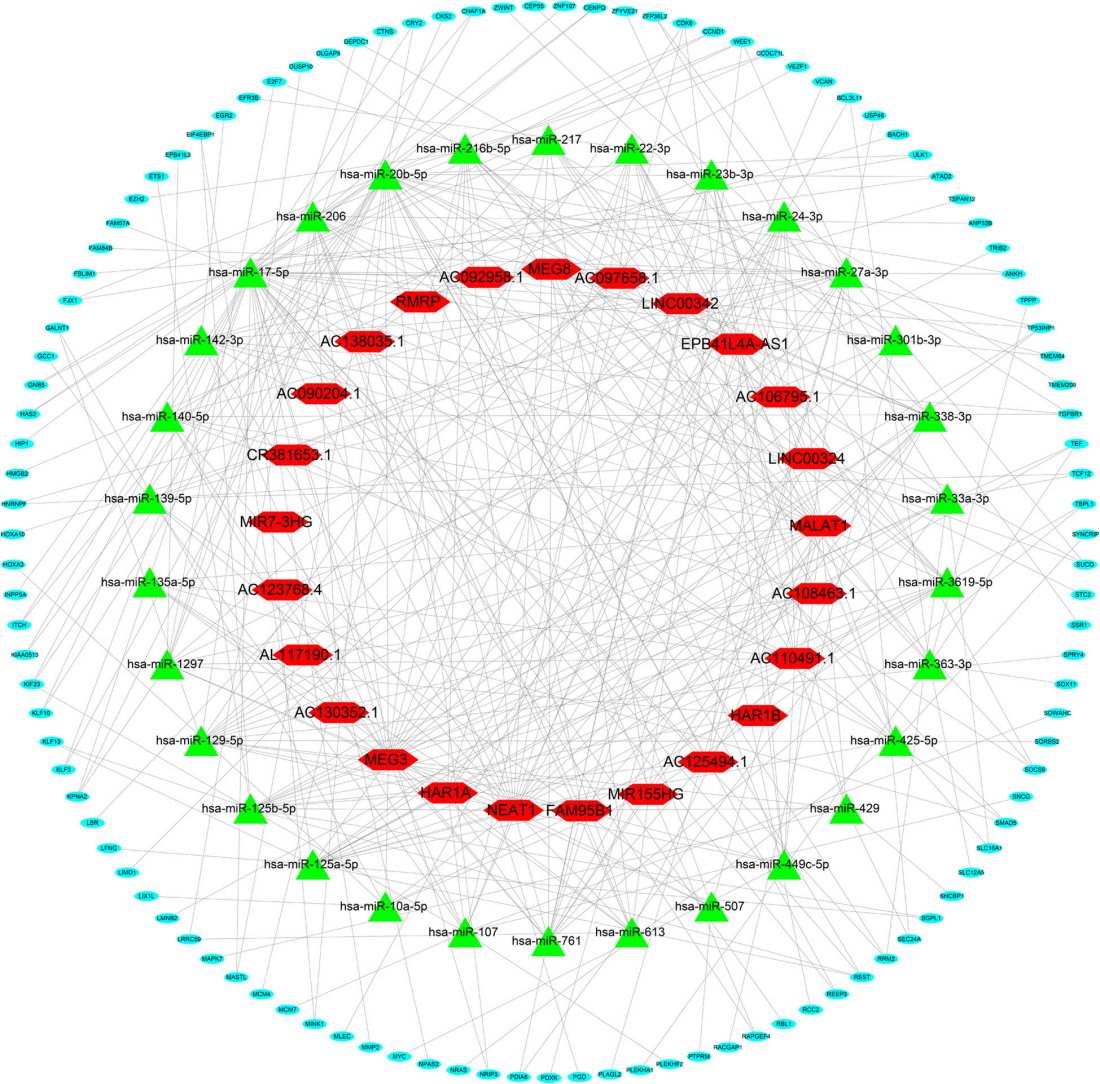
The ceRNA network of lincRNA, miRNA and mRNA. Notes: Red hexagon denotes lincRNA, green triangle represents miRNA, and cyan ellipse represents mRNA

Through WGCNA analysis of lincRNAs, we obtained 2206 lincRNAs in the turquoise module and then integrated the analysis with 339 DElincRNAs to obtain 251 overlapping lincRNAs. Further, using the miRcode online tool, 251 lincRNAs were used to predict miRNAs. Additionally, using the miRDB, TargetScan, and miRTarBase datasets, the predicted miRNAs were used to obtain the corresponding mRNAs. By analysing the predicted mRNAs, WGCNA key module mRNA (TCGA and GTEx, GEO), and DEmRNA, we obtained 111 mRNAs (24 down-regulated and 87 up-regulated mRNAs). The predicted miRNAs and 251 lincRNAs were analysed using the 111 mRNAs, and 25 lincRNAs and 30 miRNAs were finally obtained. Subsequently, we used Cytoscape version 3.7.2 software to build a lincRNA-miRNA-mRNA ceRNA network (Fig. 5).

### Functional enrichment analysis

**Fig. 6:**
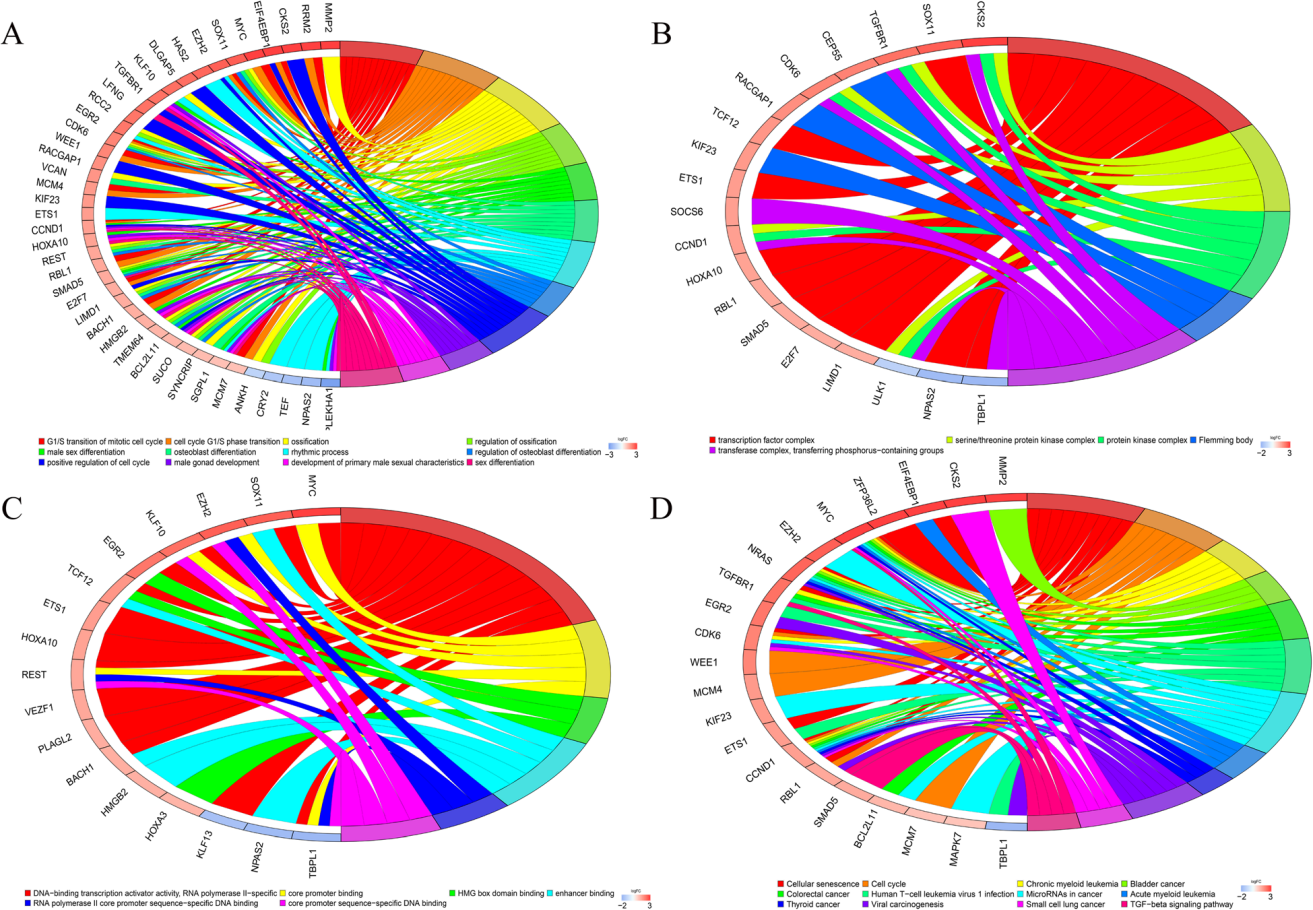
GO and KEGG analysis. **a**, The relationship between genes and GO terms of biological process. **b**, The relationship between genes and GO terms of cellular component. **c**, The relationship between genes and GO terms of molecular function. **d**, Chord plot indicates the relationship between genes and KEGG pathways. GO, Gene Ontology; KEGG, Kyoto Encyclopedia of Genes and Genomes

A functional enrichment analysis was performed on the 111 mRNAs obtained previously. The biological processes of enrichment were mainly cell cycle, ossification, and sex differentiation (Fig. 6a). The cellular components were concentrated in the transcription factor, protein kinase, and serine/threonine protein kinase complexes (Fig. 6b). Enriched molecular function was mainly involved in DNA-binding transcription activator activity, RNA polymerase II-specific core promoter binding, and HMG box domain binding (Fig. 6c). KEGG pathway analysis showed that the genes were associated with cellular senescence, cell cycle, multiple cancers, miRNA in cancer, and TGF-beta signalling pathway (Fig. 6d).

### Construction of prognostic models

**Fig. 7:**
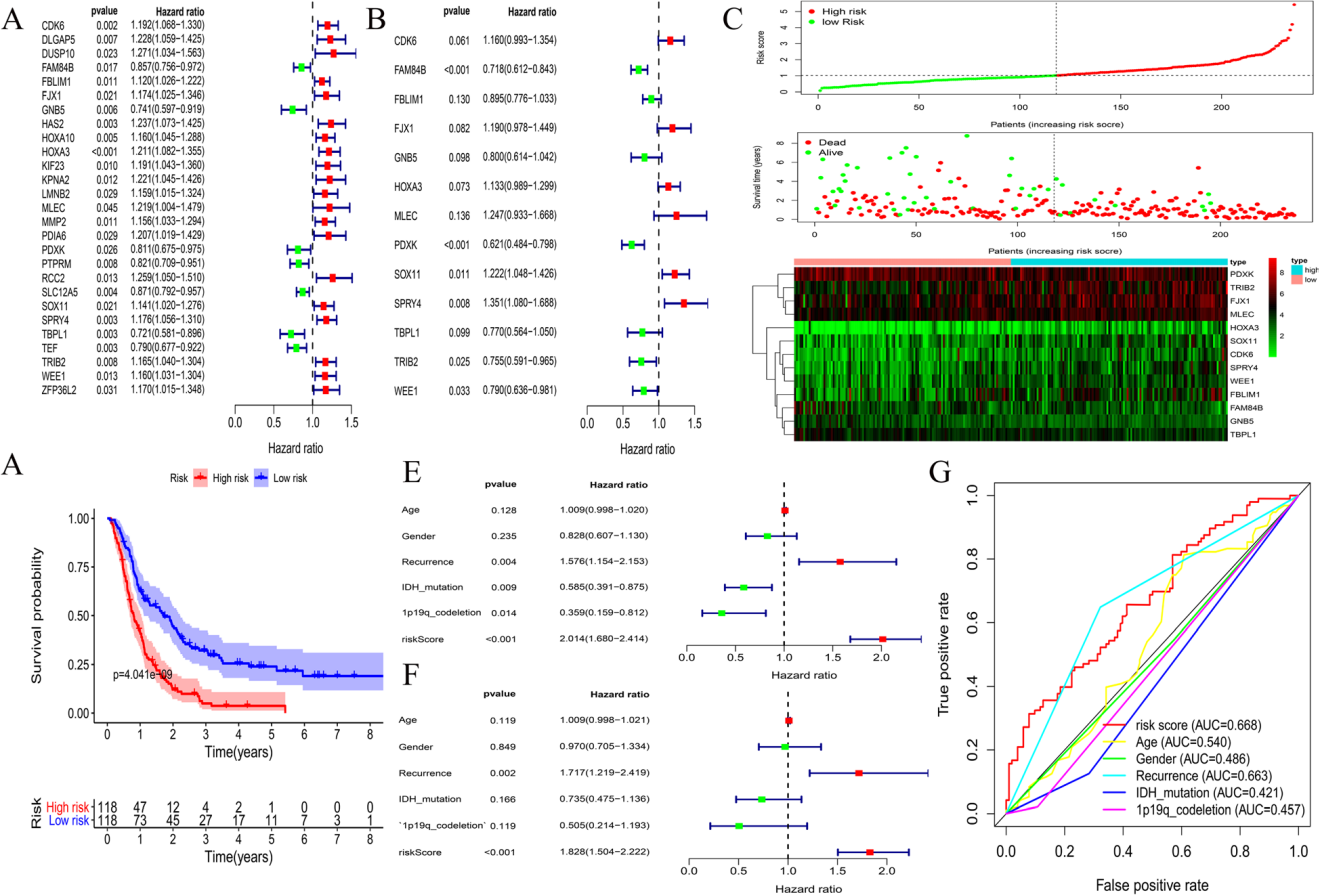
13-lincRNA prognosis model establishment and verification. **a**-**b**, Univariate Cox regression analysis and multivariate Cox regression analysis were used to construct prognostic models. **c**, Correlation between the prognostic signature and the overall survival of patients. The distribution of risk scores (upper), survival time (middle) and lincRNA expression levels (below). The black dotted lines represent the median risk score cut-off dividing patients into high- and low-risk groups. The red dots and lines represent the patients in high-risk groups. The green dots and lines represent the patients in low-risk groups. **d**, Kaplan-Meier survival curves of overall survival among risk stratification groups. **e**, Univariate Cox regression analyses of clinical factors associated with overall survival. **f**, Multivariate Cox regression analyses of clinical factors associated with overall survival. **g**, ROC curve related to clinical factors

Among the CGGA GBM samples, complete survival information was available for 236 samples, and all clinical information was available for 198 samples. Univariate Cox regression analysis was performed on the 111 mRNAs previously obtained, and 27 mRNAs related to survival time were obtained (*p* < 0.05) (Fig. 7a). Multivariate Cox analysis was performed using 27 mRNAs, and a Cox proportional hazards regression model of GBM patients containing 13 mRNAs was constructed (Fig. 7b). Based on the median risk score, all patients were divided into two groups (high-risk and low-risk groups). Figure 7c shows the survival status, survival time, and mRNA expression levels of patients. Survival analysis showed that patients in the low-risk group survived longer than those in the high-risk group (*p* < 0.001) (Fig. 7d). The univariate Cox regression analysis showed that recurrence (*p* = 0.004), isocitrate dehydrogenase (IDH) mutation (*p* = 0.009), 1p19q codeletion (*p* = 0.014), and risk score (*p* = 0.001) were predictors (Fig. 7e). Moreover, multivariate Cox regression analysis confirmed that recurrence (*p* = 0.002) and risk score (*p* < 0.001) were independent risk factors (Fig. 7f). The risk score and recurrence AUCs of the nomogram were 0.668 and 0.663, respectively (Fig. 7g).

### CeRNA network in GBM

**Fig. 8:**
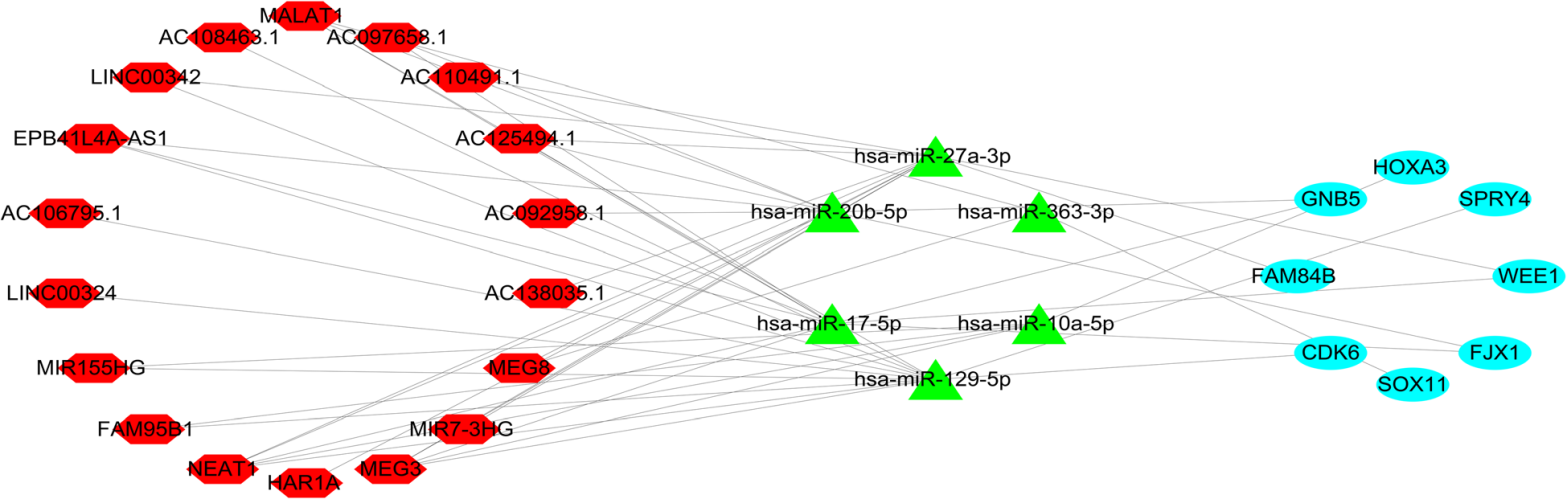
Construction of a ceRNA network including 8 mRNAs, 6 miRNAs, and 18 lincRNAs. Notes: Red hexagon denotes lincRNA, green triangle represents miRNA, and cyan ellipse represents mRNA

Using the 13 mRNAs obtained from the previous multivariate COX regression analysis conducted to screen the ceRNA network, a total of 14 miRNAs and 23 lincRNAs that interacted were found. We combined the previously selected 14 miRNAs and DEmiRNAs, screened out 6 overlapping miRNAs, and then screened the ceRNA network again. Finally, a ceRNA network containing 8 mRNAs, 6 miRNAs, and 18 lincRNAs was constructed (Fig. 8).

### GSEA reveals the close relationship between lincRNA and GBM

**Fig. 9:**
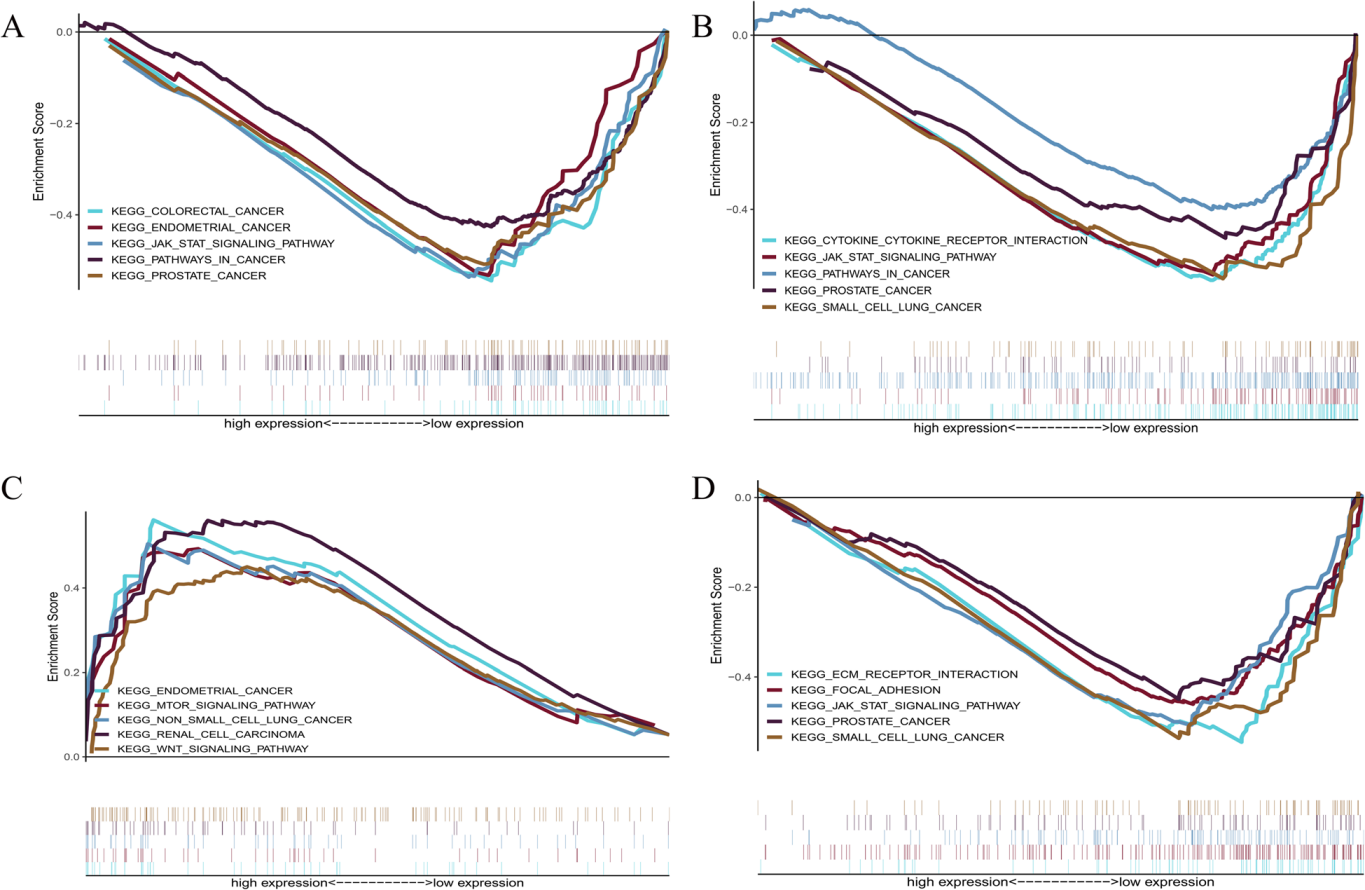
Gene set enrichment analysis (GSEA). **a**, *MEG3*; **b**, *MIR7-3HG*; **c**, *FAM95B1*; **d**, *EPB41L4A-AS1*. *P* < 0.05

Using | log2FC | ≥2 as a condition to screen 18 kinds of lincRNA, we obtained seven of them: *MALAT1*, *MEG3*, *NEAT1*, *MIR7-3HG*, *FAM95B1*, *EPB41L4A*-*AS1*, and *AC125494*.*1*. Among them, extensive research has been conducted on *MALAT1* and *NEAT1*. Thus, we selected *MEG3*, *MIR7*-*3HG*, *FAM95B1*, and *EPB41L4A*-*AS1* in 248 CGGA samples for GSEA. As shown in Fig. 8, the GSEA results revealed that these lincRNAs were closely related to cancer. *MEG3* showed potential effects on prostate cancer, colorectal cancer, and cancer pathways (Fig. 9a), *MIR7-3HG* demonstrated potential role in small cell lung cancer, prostate cancer, and cancer pathways (Fig. 9b), *FAM95B1* was closely related to endometrial cancer, non-small cell lung cancer, renal cell carcinoma, and MTOR signalling pathway (Fig. 9c), while *EPB41L4A-AS1* was associated with small cell lung cancer, prostate cancer, and JAK/STAT signalling pathway (Fig. 9d). AC125494.1 is not found in the CGGA data.

## Discussion

GBM, which is the most common type of glioma, accounts for about 15% of all brain tumours. It has been reported that there are about three GBM patients per 100,000 people, and the average survival time of patients is only approximately 12–18 months. The 5-year and 10-year survival rates are approximately 5.5 and 2.9%, respectively [28, 29]. The low recovery rate and poor survival time of GBM patients may be related to the lack of efficient therapeutic targets. Therefore, it is of great significance to find new biomolecular markers and therapeutic targets of GBM. Currently, many scholars and research institutions focus on the role of non-coding RNAs in tumours, and several molecules have been significantly associated. Studies have shown that many lincRNAs can act as oncogenes or tumour suppressor genes in cancer. In recent years, ceRNA network-related research has gained intense attention. In a ceRNA network, lincRNAs can competitively bind miRNAs to regulate the expression of target mRNAs. Additionally, several studies have shown that ceRNA network is closely related to the occurrence and development of cancer and may be valuable in predicting the prognosis of patients.

In this study, we used WGCNA to find key modules related to GBM pathogenicity and combined differentially expressed genes for analysis. Based on the lincRNA-miRNA-mRNA interaction, a ceRNA network of GBM patients was preliminarily constructed, and 111 mRNAs in the network were functionally enriched. The results showed that these mRNAs have potential roles in cell cycle, various cancers, miRNA in cancer, DNA-binding transcriptional activator activity, TGF-β signalling pathway, transcription factor complex, RNA polymerase II specificity, etc. Further, univariate Cox regression and multivariate Cox regression analyses were used to construct a Cox proportional hazards regression model with 13 key genes: *CDK6*, *FAM84B*, *FBLIM1*, *FJX1*, *GNB5*, *HOXA3*, *MLEC*, *PDXK*, *SOX11*, *SPRY4*, *TBPL1*, *TRIB2*, and *WEE1*. We calculated the risk score of patients, and verified that the prognostic model is highly accurate. In addition, the nomogram based on the risk score has the highest AUC value. We combined DEmiRNA and 13 mRNAs to screen the ceRNA network. Finally, 8 mRNAs, 6 miRNAs, and 18 lincRNAs were used to construct a ceRNA network. We selected four lincRNAs, including *MEG3*, *MIR7*-*3HG*, *FAM95B1*, and *EPB41L4A*-*AS1*, and predicted their functions using the GSEA software. Our previous studies have shown that there are few studies on these four lincRNAs and GBM. These lincRNAs were shown to be closely related to a variety of cancers. Among them, *MEG3* and *MIR7*-*3HG* are related to cancer pathways, while these two and *EPB41L4A*-*AS1* all regulate the JAK/STAT signalling pathway.

*MALAT1* and *NEAT1* have been widely studied non-coding RNAs, and some studies have demonstrated that they have clear correlations with various cancers such as hepatocellular carcinoma and lung cancer [30–33]. It has also been reported that *MALAT1* can be used as ceRNA of miR-199a to promote the expression of *ZHX1*, which in turn can regulate the proliferation of GBM cells [34]. The ceRNA effect between *NEAT1* and miR-194-5p is related to the angiogenesis of glioma [35].

*MEG3*, also known as gene trap locus 2, is an imprinted gene located at 14q32 [36]. It is a new type of tumour suppressor that plays a role in various tumours, such as ovarian and bladder cancers [37, 38]. Studies have shown that *MEG3* is under-expressed in gliomas, and its over-expression has a significant inhibitory effect on the proliferation and migration of glioma cells, while promoting its apoptosis and autophagy, and inhibiting the PI3K/AKT/mTOR signalling pathway [39, 40]. *MEG3* can act as a ceRNA for miR-19a, thereby exerting an inhibitory effect on glioma [41]. The present study also predicted that *MEG3* is potentially valuable in multiple tumours and cancer pathways.

According to previous reports, *MIR7-3HG* is associated with tumour progression and is highly expressed in endometrial cancer. Bioinformatics analysis by Wang et al. showed that its high expression in breast cancer is significantly related to the survival time of patients [42, 43]. It has also been reported that this gene can be used as ceRNA to up-regulate the expression of *PEG10* by sponged miR-27a-3p, and thus plays a role in retinoblastoma [44]. *EPB41L4A-AS1*, located in the 5q22.2 region of the human genome, is an induced gene of p53 and pGC-1α, can regulate glycolysis and glutamine metabolism, and plays an important role in cancer metabolic reprogramming [45]. It is highly expressed in colorectal cancer tissues and is involved in the proliferation, invasion, and migration of colorectal cancer cells [46]. Additionally, *FAM95B1* has been shown to be associated with thyroid cancer [47]. Limitations of our study include the lack of cell or animal experiments and its retrospective nature. Subsequently, we hope to verify our results by conducting cell-based experiments.

## Conclusion

Conclusively, in this study, we constructed the lincRNA-miRNA-mRNA ceRNA network of GBM, which may be involved in its molecular regulation, and identified four lincRNAs with potential roles in the tumour. To our knowledge, three of these lincRNAs, namely *MIR7*-*3HG*, *FAM95B1*, and *EPB41L4A*-*AS1*, are novel potential therapeutic targets for GBM, as there are no previous related studies. Further, we created a prognostic model containing 13 genes, which may serve as a reliable prognostic indicator of GBM. Limitations of our study include the lack of cell or animal experiments and its retrospective nature. Subsequently, we hope to verify our results by conducting cell-based experiments. However, the present study is expected to facilitate in-depth understanding and study the molecular mechanism of GBM, and provide new insights into targeted therapy and prognosis of tumours.

## Data Availability

The data comes from TCGA, GTEx, GEO, CGGA databases, which are all public open platforms.

## Abbreviations

LincRNA: Long intergenic non-coding RNA
ceRNA: Competitive endogenous RNA
GBM: Glioblastoma
TCGA: The cancer genome atlas
NCBI: National center for biotechnology information
GEO: Gene expression omnibus
CGGA: Chinese glioma genome atlas
GTEx: Genotype tissue expression
WGCNA: weighted gene co-expression network analysis
GO: Gene ontology
KEGG: Kyoto encyclopedia of genes and genomes
GSEA: Gene set enrichment analysis
